# An infrared-transparent flexible glass for adaptive optics

**DOI:** 10.1038/s41377-026-02409-z

**Published:** 2026-07-30

**Authors:** Saihui Li, Linling Tan, Jianqiang Ma, Shiliang Kang, Chengwei Gao, Shixun Dai, Changgui Lin

**Affiliations:** 1 Laboratory of Infrared Materials and Devices, The Research Institute of Advanced Technologies, Ningbo University, Ningbo, 315211 China; 2 Key Laboratory of Photoelectric Detection Materials and Devices of Zhejiang Province, Ningbo, 315211 China; 3 Engineering Research Center for Advanced Infrared Photoelectric Materials and Devices of Zhejiang Province, Ningbo, 315211 China; 4 College of Mechanical Engineering and Mechanics, Ningbo University, Ningbo, 315211 China

**Keywords:** Optical materials and structures, Adaptive optics, Mid-infrared photonics

## Abstract

Transparent, flexible inorganic-organic composites are essential for next-generation adaptive optics, bio-integrated sensing, and reconfigurable photonics. Despite progress in hybrid material design, achieving a material that is simultaneously flexible and transparent across a broad infrared spectrum remains a fundamental challenge. Here, we report a S_60_Se_40_ chalcogenide glass designed with a chain-ring dual-network architecture that overcomes this classic limitation. By integrating a dynamic covalent network of physical cross-links with heavy chalcogen elements (S, Se), this dual-network design suppresses multi-phonon absorption and ensures broadband transparency up to 21 μm, combining these optical properties with an ultralow Young’s modulus (*E* ≈ 0.0037 GPa), extreme tensile strain (~650%), and high elastic recovery (~80%). The material further exhibits autonomous self-healing and shape-memory behavior at room temperature. We demonstrate its applicability through IR deformable lenses with tunable focal length and real-time aberration correction, showcasing capabilities in adaptive imaging and wavefront control. This work provides a versatile material platform that bridges the gap between glass-like optical performance and polymer-like mechanics, opening avenues for soft infrared photonics and reconfigurable optical systems.

## Abstract (web summary)

A chain-ring dual-network chalcogenide glass reconciles flexibility with IR transparency, overcoming the trade-off for deformable adaptive optics.

## Graphical abstract

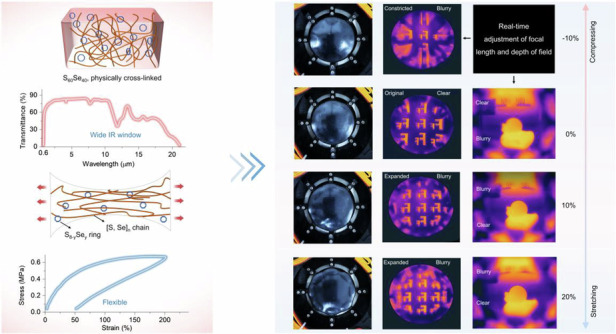

## Introduction

The combination of inorganic glass networks with organic components offers a promising route toward transparent, flexible composites, merging the excellent processability and surface quality of bulk glasses with the biocompatibility, tunable mechanics, and versatile optoelectronic responses of organic phases^1–4^. Such hybrid materials have long been a critical unmet need for emerging technologies—including adaptive optical systems^2,3,5^, steerable bioimaging and sensing^5,6^, reconfigurable photonic devices^7^, and dynamically tunable industrial metrology platforms^3,8^. However, despite progress in inorganic-organic composites design, achieving simultaneous broad-spectrum optical transparency (particularly in the infrared (IR) spectral region) and high mechanical flexibility remains a fundamental challenge^1,9,10^. This limitation stems from an inherent property conflict: IR transmission requires a rigid three-dimensional network composed of heavy elements to suppress vibration absorption^11,12^, whereas mechanical compliance typically arises from soft, dynamically bonded molecular chains that exhibit strong IR absorption^13^. This trade-off is clearly demonstrated in conventional optical materials. For instance, germanium and chalcogenide crystals transmit well beyond 20 μm but exhibit high Young’s moduli (*E* ≈ 100 GPa)^14,15^. In contrast, soft polymers such as polyurethane (PU) achieve moduli as low as 0.01 GPa but become opaque beyond 2.5 μm^1,16^. Recent strategies, including modified chalcogenides with improved flexibility (*E* ≈ 20 GPa), ultrathin polymer films^17,18^, and novel hybrid sulfur polymers^1,9,19^, still fall short. They have failed to effectively combine IR transparency with the desired mechanical flexibility, i.e., “IR-transparent yet flexible”.

IR adaptive optics technology accentuates the urgency of this materials challenge. The technology is rapidly expanding beyond astronomical observation into critical fields such as space laser communication^20^, ground-based high-resolution surveillance^21^, laser precision machining^22^, and biological microscopic imaging^23^. The core requirement of these applications lies in the real-time, precise correction of dynamic wavefront aberrations to maintain system performance near the diffraction limit. Current implementations, however, face fundamental limitations. Transmissive deformable lenses (DLs) based on flexible organics (e.g., PDMS, hydrogels)^24–26^ or liquid components (e.g., water, silicone oil)^27,28^ are largely restricted to the visible spectrum due to strong IR absorption. While reflective deformable mirrors based on micro-electromechanical systems or thin films provide an alternative for IR wavefront correction, their complex multi-stage optical configurations limit them to large-scale systems such as astronomical telescopes, with spatial resolution further constrained by practical limitations in actuator density and arrangement^21,29–31^. These persistent challenges across both transmissive and reflective approaches highlight the need for a new class of “IR-transparent yet flexible” materials that could enable compact, high-performance adaptive optics systems operating across the IR spectrum.

Here, we present an S_60_Se_40_ IR ultra-flexible chalcogenide (IR-FCh-60) glass that overcomes the IR transparency-flexibility trade-off through a chain-ring dual-network (DN) architecture. Our approach leverages the synergistic coexistence of two distinct structural motifs: a covalent chain network composed of heavy-weight elements that ensures broadband IR transparency, and physically cross-linked ring domains that enable polymer-like mechanical compliance. This structural design simultaneously achieves an ultralow Young’s modulus (*E* ≈ 0.0037 GPa), extreme tensile strain (~650%), and broad IR transmission up to 21 μm. The material further demonstrates autonomous self-healing, shape memory, and high strain recovery (~80%) under room temperature, thereby realizing the previously inaccessible “IR-transparent yet flexible” property. As a functional demonstration, we fabricate IR-DLs capable of focal-length tuning and aberration correction, highlighting the material’s potential for IR adaptive optics, biomedical imaging systems, and next-generation space technologies.

**Fig. 1:**
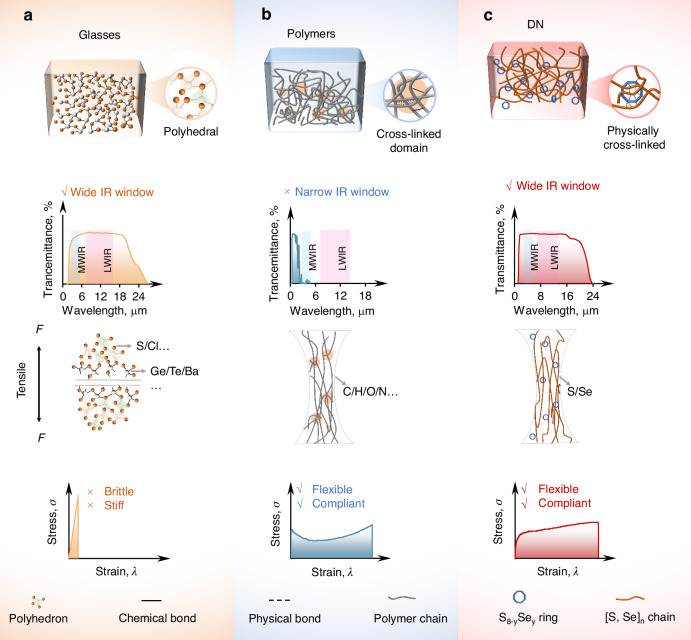
DN architecture of an IR-transparent flexible glass. Schematic comparison of material design and the resultant properties: **a** stiff IR-transparent glasses with rigid three-dimensional covalent networks; **b** soft polymers with dynamically cross‑linked chains; **c** S_60_Se_40_-based DN chalcogenide glass, which integrates physically cross-linked S_8−*y*_Se_*y*_ rings with polymeric [Se, S]_*n*_ chains. Representative transmission spectra and stress-strain curves illustrate how the DN architecture achieves both a broad IR transmission window and a compliant, hyperelastic mechanical response

This DN design is inspired by the complementary properties of glassy and polymeric materials^12,32^. Conventional inorganic glasses achieve IR transparency through rigid three-dimensional covalent networks of heavy elements that effectively suppress molecular vibrations, yet their lack of reversible bonding leads to brittle fracture under strain (Fig. 1a)^11^. By contrast, polymers attain flexibility through dynamically cross-linked networks featuring chemical cross-links with high degrees of freedom and physical cross-links with weak interactions; however, their light-element bonds (e.g., C–H, O-H) introduce strong IR absorption (Fig. 1b)^10,33^. To synergistically combine these attributes, we developed a chain-ring DN architecture based on heavy chalcogen elements (Fig. 1c).

The design incorporates two key features: (i) covalent [Se, S]_*n*_ chains that suppress multi-phonon absorption to ensure broad IR transparency, and (ii) physically cross-linked ring domains interwoven with covalent chains form a dynamic topological network. In this network, ring and chain segments exhibit high entanglement and low inter-chain friction, while the chain domains behave as load-bearing units under moderate stress. The physical bonds between rings and chains are substantially weaker than the covalent intra-chain bonds, allowing energy dissipation through reversible slippage under high stress while maintaining structural integrity via multi-domain connectivity. Moreover, S–S, S–Se, and Se–Se bonds throughout the network act as dynamic covalent motifs, conferring autonomous self-healing capability. Within this hierarchical architecture, the covalent network preserves macroscopic IR optical properties and structural stability, while the dynamic ring domains collectively provide ultralow modulus, large strain, high recovery, and self-healing functionality through reversible bond reorganization.

## Results

### Unique mechanical-optical coupling in IR-FCh-60 glass

**Fig. 2:**
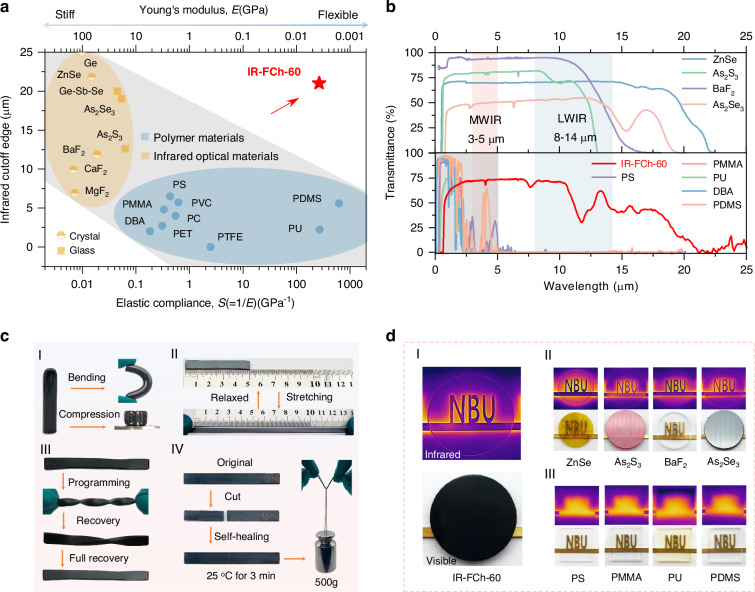
IR-FCh-60 glass with polymer-like flexibility and glass-like IR transparency compared with conventional optical materials. **a** Property map showing that IR-FCh-60 glass overcomes the conventional IR transparency-flexibility trade-off (gray band), which restricts typical optical materials to being either “IR transparency and stiff” or “strong IR absorption and flexible.” **b** Transmission spectra (2 mm thickness) of IR-FCh-60 compared with standard IR materials (ZnSe, As₂S₃, BaF_2_, As₂Se₃) and optical polymers (PS, PMMA, PU, DBA, PDMS). IR-FCh-60 exhibits a long-wave IR cutoff (>20 μm) and an ultra-broad transmission window of 20.38 μm. In contrast, polymers display strong characteristic absorption bands across the MWIR to LWIR, leading to narrow transmission windows and short cutoff wavelengths (<6.5 μm). **c** Visual evidence for mechanical behavior demonstrating polymer-like flexibility: (I) bending and compression, (II) stretching, (III) shape memory, (IV) self-healing (Supplementary Movies S1–S5). These behaviors resemble those of organic elastomers (e.g., PU and PDMS), and contrast with the brittle nature of conventional IR optical materials. A cylindrical sample (Φ9 mm × 70 mm) and a rectangular strip (length × width × thickness = 50 mm × 5 mm × 1.5 mm) were used. **d** Visual evidence for the IR transmission performance of IR-FCh-60 glass, like conventional IR optical materials, whereas organic substances exhibit complete opacity in the same spectral regime. (I) IR-FCh-60, (II) typical IR optical materials (ZnSe, As_2_S_3_, BaF_2_, and As_2_Se_3_), and (III) optical polymers (PS, PMMA, PU, and PDMS) were shown. Top row: IR images; bottom row: visible-light images. All 2 mm-thick samples were placed against a heated brass background consisting of the characters “NBU”

As illustrated in Fig. 2a and Supplementary Fig. S1a, IR-FCh-60 glass with amorphous characteristics breaks through the well-known trade-off between IR transparency and flexibility that limits conventional optical materials (indicated by the gray band of Fig. 2a). It exhibits an ultra-long IR cutoff edge of 21 μm, comparable to that of Ge and chalcogenide crystals/glasses, combined with a polyurethane-like ultralow Young’s modulus of 0.0037 GPa (corresponding to an ultrahigh elastic compliance of 270 GPa^−1^). This unique combination of properties places IR-FCh-60 within the highly desirable “IR-transparent yet flexible” region (red five-pointed star in Fig. 2a).

Figure 2b demonstrates that IR-FCh-60 glass exhibits high IR transparency with an ultra-long cutoff edge of 21 μm. Notably, this 21 μm cutoff edge not only far exceeds the relatively short cutoff edge (2.2–6.5 μm) of organic optical materials, such as PMMA, PS, PDMS, PU, but also surpasses those (6–13 μm) of many typical IR materials such as fluoride crystals and commercial chalcogenide glasses. The ultra-broad transmission window spans from 0.62 to 21 μm (bandwidth ≈ 20.38 μm), fully encompassing the two key atmospheric windows at 3–5 μm and 8–14 μm (Fig. 2b and Supplementary Fig. S1b, c). Notably, the material maintains excellent IR transparency even after 1 year of storage in air, and exhibits only minor fluctuations in transmission following mechanical deformation (Supplementary Fig. S1d, e), further demonstrating its robust optical stability for practical applications.

Beyond its exceptional optical performance, IR-FCh-60 exhibits flexibility, sustaining large bending, compression, and tensile deformations at room temperature without fracture (Fig. 2cI, II, and Supplementary Movies S1–S3). Moreover, it demonstrates intrinsic self-recovery behavior, including shape memory and autonomous self-healing: the material can be programmed into a spiral shape under torsion and fully recovers upon release (Fig. 2cIII and Supplementary Movie S4). Remarkably, after being severed, two segments of IR-FCh-60 can self-heal without thermal activation and regain mechanical strength sufficient to support a 500 g weight (Fig. 2cIV and Supplementary Movie S5). This self-healing capability exceeds that of PU (which can only sustain its own weight) and PDMS (which lacks self-healing capability at room temperature) (Supplementary Fig. S2a, b and Supplementary Movie S5). More specifically, after only 3 min of self-healing, IR-FCh-60 rapidly recovered a large tensile strain of up to 151%, while its fracture stress reached 81.8% of the original value. In contrast, PU exhibited limited recovery, with a tensile strain of 4.4% and a fracture stress of 4.6% of the original value (Supplementary Fig. S2c, d). This unique integration of extreme stretchability, instant shape recovery, and robust self-healing bridges the gap between organic elastomers and high-performance inorganic materials, which has long been desired by many emerging technologies.

Figure 2d shows visual evidence for the IR transmission performance of the IR-FCh-60 glass material. Its imaging quality matches that of high-cost transmissive materials such as fluoride and sulfide crystals/glasses, while being distinctly superior to conventional organic materials (e.g., PS, PMMA, PU, PDMS), which are largely opaque in the IR region^1,34^.

**Fig. 3:**
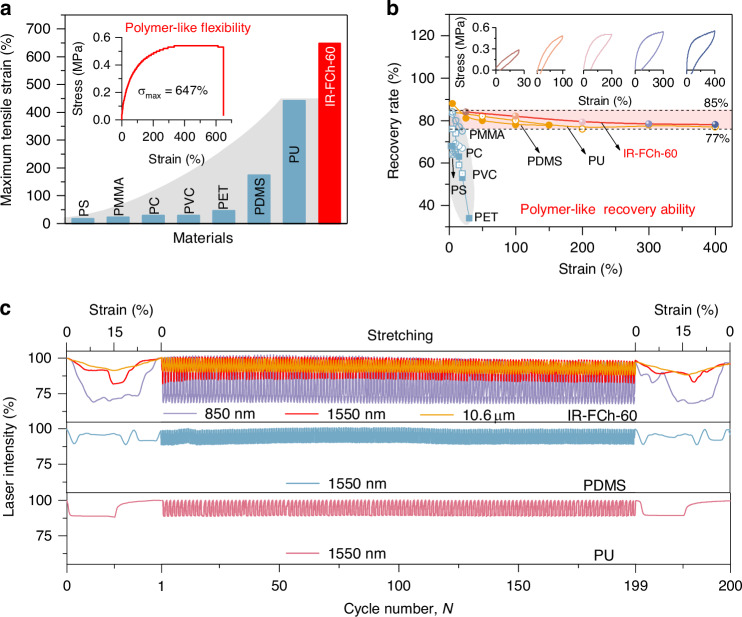
IR-FCh-60 glass shows polymer-like flexibility together with self-recovery capability and optomechanical stability during cyclic stretching. **a** Maximum tensile strain of IR-FCh-60 glass as compared with existing optical polymers (inset: representative stress-strain curve of IR-Ch-60) (Supplementary Fig. S3a, b). **b** High recovery rate (~80%) of the IR-FCh-60 across a wide tensile strain range (25–400%), contrasting with conventional optical polymers, including high elastic modulus variants (PC, PS, PMMA, PET) and ultralow modulus systems (PDMS, PU) (Supplementary Fig. S3c-i). Inset: stress relaxation behavior of IR-FCh-60 at varied strains. The recovery rate is defined as the ratio of the strain range with non-zero stress during the recovery phase of the tensile recovery curve to the maximum tensile strain within the same cycle. **c** Transmission during cyclic stretching (0–15% strain) for polymer-like IR-FCh-60 (at 850 nm, 1550 nm, and 10.6 µm), PDMS, and PU (at 1550 nm) as a function of cycle elongation. Cycles 1 and 200 show deformation-dependent attenuation, while cycles 2–199 demonstrate repeatable behavior

The ultralow modulus endows IR-FCh-60 with a large tensile strain of 647%, substantially surpassing that of traditional organic optical materials (high-modulus PC, PS, PMMA, PET: <50%; ultralow modulus PDMS and PU: 170 and 410%, respectively)^35,36^, as shown in Fig. 3a, Supplementary Figs. S2g and S3a, b. Furthermore, IR-FCh-60 glass can withstand compressive strain greater than 50% and bending strain over 15% without fracture (Supplementary Fig. S2e, f and Supplementary Movies S1 and S2). Such a level of tensile strain enables large-range focal tuning, which is essential for IR adaptive optics^24,37^.

The polymer-like IR-FCh-60 exhibits self-recovery at ambient temperature, with a strain recovery ratio of approximately 80%, rivaling that of shape-memory polymers (Fig. 3b). Crucially, this high recovery ratio is maintained across a broad tensile strain range from 25 to 400%, comparable to flexible organic materials such as PDMS and PU. This contrasts sharply with high-modulus thermoplastics (PMMA, PC, PET, PS), which suffer from significant recovery loss under strain due to irreversible plastic deformation (Supplementary Fig. S3c-i). The combination of ultralow modulus, extreme flexibility, and strain-invariant self-recovery fulfills the stringent requirements for deformation-tolerant IR adaptive optics^38^.

To quantify the strain-dependent transmission of the polymer-like IR-FCh-60 glass, we monitored transmitted light intensity at 0.85, 1.55, and 10.6 µm during cyclic stretching (0–15% strain; Fig. 3c). Under identical conditions, comparative studies with PDMS and PU revealed reversible transmission attenuation upon elongation over 200 cycles. The IR-FCh-60 glass maintained nearly constant transmittance across wavelengths with minimal hysteresis, demonstrating superior optical resilience under repeated mechanical perturbation compared to polymeric counterparts^39^. This exceptional optomechanical stability enables real-time focal tuning in deformable IR optics without signal degradation.

### Strain-driven dual-network (DN) reconfiguration of IR-FCh-60 glass

**Fig. 4:**
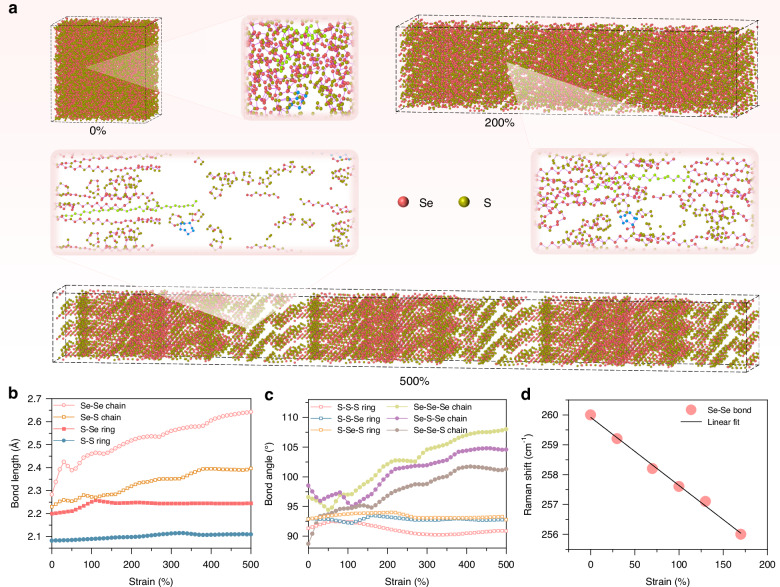
Strain-driven structural evolution of polymer-like IR-FCh-60 glass. **a** Molecular dynamics snapshots and cross-sectional views (partially enlarged views) at 0, 200, and 500% tensile strain. Polymeric [Se, S]_*n*_ chains and eight-membered Se_*y*_S_8−*y*_ rings are highlighted in green and blue, respectively, to track structural evolution during deformation. Under uniaxial elongation, the coiled polymeric chains progressively extend toward linear configurations, while the eight-membered rings largely retain their structural integrity. Evolution of **b** bond length and **c** bond angle in chain versus ring during elongation. Chains show significant covalent bond elongation, especially Se–Se bond elongation (>0.3 Å increase) and angular distortion (>10° increase), whereas rings exhibit minimal changes in bond parameters (<0.05 Å variation). **d** Raman shift of the Se–Se bond as a function of strain. The redshift (Δ*ν* = −4 cm^−1^ at 170% strain) confirms strain-induced bond elongation and angular expansion within the chains, which reduces vibrational frequency by increasing interatomic distances^49^

Figure 4a identifies two topological structures in this untraditional polymer-like material: polymeric [Se, S]_*n*_ chains and eight-membered S_8−*y*_Se_*y*_ rings (0 ≤ *y* ≤ 8)^40^. These structural motifs form a DN architecture in IR-FCh glasses, analogous to that in DN polymers^41^. This organization fundamentally differentiates IR-FCh glasses from conventional IR optical materials, which typically consist of either long-range ordered crystalline lattices or disordered networks based on pyramidal/tetrahedral units^11,18^. Crucially, the twofold chalcogen coordination of the rings prevents covalent bonding to the chains, enabling dynamic physical crosslinking. Strategic modulation of the DN parameters allows broad tuning of mechanical properties (Fig. 2c, Supplementary Fig. S2), facilitating customizable flexibility, recovery, self-healing, and shape-memory behaviors for functional integration.

Molecular dynamics simulations reveal the structural evolution of IR-FCh-60 glass under tensile strain (0–500%), illustrating dynamic reorganization within the atomic configuration and molecular packing of both the [Se, S]_*n*_ chains and Se_*y*_S_8−*y*_ rings (Fig. 4a). Under uniaxial stretching, the initially homogeneous and close-packed structure evolves into alternating inhomogeneous domains: ring-rich sparse regions and chain-rich dense areas. This molecular-scale phase separation is driven by chain entanglements and weak inter-ring interactions. Cross-sectional analysis shows progressive extension and slippage of the initially curled polymeric chains, while the eight-membered rings undergo sliding deformation with minimal structural change. Acting as mobile topological cross-linkers, the rings facilitate rapid stress redistribution through cooperative slippage and migration between chains and rings. Concurrently, extended chain length and strong covalent bonds prevent fracture. This mechanism facilitates ultrahigh tensile strain (~650%) without failure through the combined actions of chain extension and controlled slippage. This deformation mode parallels that observed in physically cross-linked DN polymers^32,42^. Notably, structural heterogeneity (chain stretching and slippage, ring displacement) diminishes with reduced sulfur content (Supplementary Fig. S4a), due to decreased cooperative mobility of eight-membered rings.

Under increasing tensile strain, the Se–Se–Se bond lengths and angles in the polymeric chains exhibit near-linear increases (Fig. 4b, c). Conversely, the eight-membered rings display limited changes in bond parameters, constrained by strong intra-ring interactions (Fig. 4b, c). Tensile loading preferentially alters chain configurations, leading to resulting in a remarkable redshift of approximately 4 cm^−1^ in the Raman signal of Se–Se bond at 170% strain (Fig. 4d). In comparison, vibrations related to S–Se (in both chains and rings) and S–S (in rings) show negligible changes (<2 cm^−1^ in total; Supplementary Fig. S5). Reduction in sulfur content further suppresses changes in bond parameters and Raman peak displacements (Supplementary Figs. S4b–g and S5), consistent with molecular dynamics simulations that indicate restricted ring deformation under strain.

### Origin of polymer-like ultra-flexibility with IR transparency

We demonstrate that the DN structure of IR-FCh-60 glass incorporates highly mobile eight-membered Se_*y*_S_8−*y*_ rings as topological cross-linkers, interconnected with long covalent [Se, S]_*n*_ chains via physical interactions (Fig. 4a). The exclusive use of heavy atoms (S and Se) endows the material with exceptionally low phonon energy, as evidenced by a characteristic Se–Se absorption peak at 255 cm^−1^ (Supplementary Fig. S5a). This suppresses multi-phonon processes, thereby extending the long-wavelength IR cutoff to ~21 μm (Fig. 2b). Meanwhile, the strong covalent bonding network shifts the short-wavelength cutoff into the visible range (~625 nm; Supplementary Fig. S1c). Together, these features yield an ultra-broad transmission window from 0.62 to 21 μm, spanning major atmospheric windows. This structural design also facilitates an ultralow Young’s modulus (~0.0037 GPa), extreme tensile strain (~650%), autonomous recovery, and self-healing capability. Network stability is maintained by robust covalent chains that resist fracture, while stress dissipation occurs via ring-mediated topological rearrangement through dynamic bond exchange. This results in a self-regulating equilibrium that underpins the material’s mechanical adaptability.

**Fig. 5:**
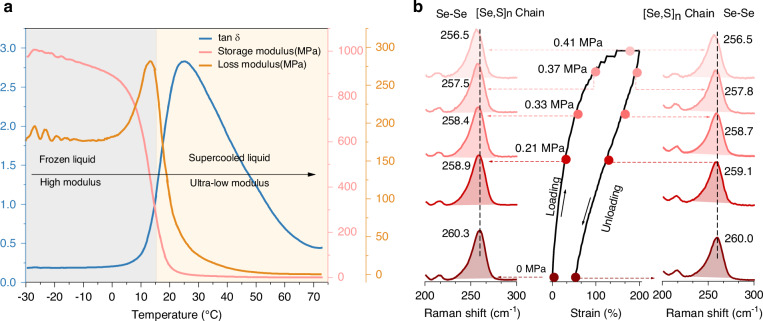
Origin of the polymer-like ultra-flexibility. **a** Dynamic mechanical analysis of IR-FCh-60 glass, showing temperature dependence of the storage modulus (*E*ʹ), loss modulus (*E*"), and tan δ (*E*"/*E*ʹ). As the temperature rises, *E*ʹ decreases significantly, while *E*" peaks at 13.5 °C, indicating the glass transition temperature (*T*_g_). The subsequent decline in modulus above *T*_g_ leads to ultralow modulus behavior at room temperature. **b** In situ Raman spectra during tensile loading (left: 0 → 0.41 MPa) and unloading (right: 0.41 → 0 MPa). The reversible shift of the Se–Se vibration frequency from 260.3 to 256.5 cm^−1^ under load, and its recovery to 260 cm^−1^ upon unloading, confirms elastic elongation of covalent bonds during deformation. Rectangular strip sample (length × width = 10 mm × 5 mm, thickness ≈ 10 µm) was used

The ultralow modulus of IR-FCh-60 glass arises from weak physical interactions between chains and rings, enabling rapid topological reorganization. Dynamic mechanical analysis (DMA) confirms that the physically cross-linked rings critically reduce the modulus: IR-FCh-60 exhibits a storage modulus ∼340-fold lower than pure Se glass and ∼230-fold lower than IR-FCh-30 at room temperature (Fig. 5a, Supplementary Fig. S6a). This structure also leads to a low glass transition temperature (*T*_*g*_ = 13.5 °C). Notably, these glasses exhibit polymer-like ductility in both “frozen liquid” and “supercooled liquid” states when intermolecular interactions are sufficiently weak. The physically cross-linked chain-ring topology thus enables simultaneous exceptional flexibility in the ultralow-modulus “supercooled liquid” state and enhanced ductility in the “frozen liquid” state (Supplementary Fig. S6b). Importantly, this physically cross-linked DN structure demonstrates that viscoelasticity in glassy materials can emerge without requiring a thermally driven transition from “frozen liquid” to “supercooled liquid” above *T*_g_—contrasting the perspective proposed by Dai et al.^43^.

The extreme tensile strain of IR-FCh glasses is primarily facilitated by the straightening and slippage of polymerized [Se, S]_*n*_ chains, which form a fracture-resistant load-bearing skeleton. Under uniaxial tension, stress is selectively channeled through the covalent chains because their strength markedly exceeds that of the physical cross-links. The resulting stress concentration prompts the ring domains to slip, serving as a key energy-dissipation mechanism within the DN architecture. Throughout deformation, dynamic breaking and reformation of physical cross-links allow continuous stress redistribution across the network. The extended length and covalent nature of the chains prevent pull-out or rupture, whereas reversible slippage at the chain-ring interfaces accommodates large strains without failure. The fracture strain increases proportionally with chain density (Supplementary Fig. S2g and Table S4), consistent with a mechanism dominated by chain extension and controlled slip.

Despite significant hysteresis inherent to its physically cross-linked DN architecture, IR-FCh-60 achieves recovery ratios of ~80% over a wide strain range (25–400%). This high recovery stems from weak physical interactions and dynamic cross-link exchange at ring sites, which reduce retractive resistance and enhance chain mobility. Accordingly, both recovery performance and resilience improve with ring density (Supplementary Fig. S2h). This trend is further supported by the mechanical behavior of compositions with different ring densities (Supplementary Fig. S2g and Table S4): IR-FCh-30 (fewer rings, more chains) exhibits a distinct yield point due to chain entanglement and scission, whereas IR-FCh-60 (higher ring fraction) shows a smooth, yield-free curve. Similarly, when tested at temperatures above their respective *T*_*g*_ (Supplementary Fig. S6c, d), IR-FCh-30 remains predominantly plastic, while IR-FCh-60 maintains strong elasticity. In situ tensile Raman spectroscopy (Fig. 5b) reveals reversible lengthening and contraction of Se–Se bonds in the chains during loading–unloading cycles (up to 200% strain). Applied stress elongates bonds, manifesting as a Raman redshift; upon unloading, the bonds revert nearly to their original state.

The self-healing capability of IR-FCh-60 at room temperature requires two concurrent conditions: high molecular mobility of the chain-ring motifs (evidenced by its sub-ambient *T*_g_) and a dynamic bond repertoire comprising disulfide (S–S)^44^, diselenide (Se–Se)^45^, and thioselenide (Se–S)^46^ bonds. At temperatures above *T*_g_, enhanced mobility promotes continuous bond exchange, enabling autonomous repair.

### Strain-programmable IR optics via polymer-like IR-FCh-60 glass

As a strain-programmable chalcogenide material that combines broad IR transparency with polymer-like flexibility, the IR-FCh-60 glass enables dynamically tunable IR optics capable of real-time aberration correction. To demonstrate this, we fabricated a plano-convex IR-DL featuring eight axisymmetric metallized actuation points and a tunable clear aperture (Supplementary Fig. S7a, b), designed for efficient collection of IR radiation across short to long wavelengths. Using fused deposition modeling followed by optical polishing, the IR-DL achieves superior surface quality in its relaxed state, with a peak-to-valley (PV) value of 1.572 μm and root mean square (RMS) roughness of 0.159 μm, outperforming existing deformable polymer lenses (PV: 1.76 μm; RMS: 0.40 μm) (Supplementary Fig. S7c, d)^26^.

**Fig. 6:**
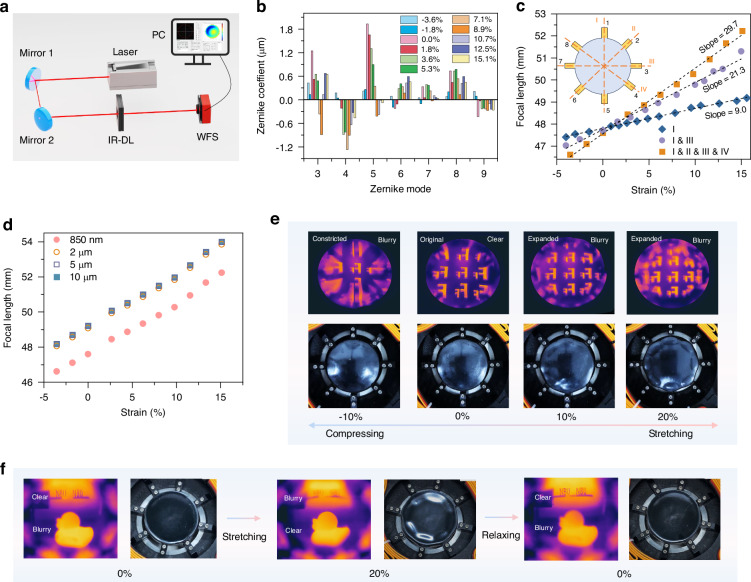
Programmable IR optics enabled by IR-FCh-60 deformable lenses. **a** Schematic of the transmitted wavefront characterization setup for an IR-DL using a custom optical bench equipped with a wavefront sensor (WFS). A collimated 850 nm laser beam passes through the IR-DL and is collected by the WFS (8 mm aperture, 1 ms exposure, 7 Hz acquisition rate). Wavefront aberrations were characterized by fitting the first 65 Zernike terms, excluding tilt components. The defocus coefficient was measured with a precision of ±0.05 µm (*λ* ∙ 12^−1^). **b** Evolution of Zernike coefficients under simultaneous quadra-axial strain (axes I–IV). **c** Focal length tuning under vectored actuation: uniaxial (I), biaxial (I + III), and quadra-axial (I–IV). Defocus was nulled at each strain by WFS displacement. Inset: Actuator numbering and axes. **d** Focal length tuning range under quadra-axial strain at 850 nm. Focal lengths derived from lens curvature and refractive index *f* = *R*/(*n* − 1), where *R* is the radius of curvature and *n* the refractive index) were extrapolated to wavelengths of 2, 5, and 10 µm. **e** Visible-light and thermal IR images of a plano-convex IR-DL under relaxed, compressive, and tensile states, showing deformation-dependent imaging evolution. **f** Real-time focal switching and depth selectivity demonstrated with a plano-concave IR-DL: the distant “NBU” object is sharply imaged in the relaxed state; focus shifts to the nearer “Duck” object under stretching; and original focus recovers upon strain release

Initial characterization using a custom optical setup (Fig. 6a) quantified strain-induced modulation of wavefront aberrations via Zernike coefficients under four-axis symmetric actuation. Simultaneous actuation across all four axes allowed selective tuning of astigmatism and defocus (Zernike terms *Z*_3_, *Z*_4_, and *Z*_5_), while ignoring piston and tilt errors (*Z*₁, *Z*₂) (Fig. 6b). The theoretical focal length derived from lens curvature and refractive index (47.8 mm) closely matched experimental measurements in the undeformed state (47.5 mm; Fig. 6c, Table S5). Focal-length tuning was achieved by applying strain along defined vectors: uniaxial (actuator I), biaxial (I and III), and quad-axial (I–IV). This approach revealed a linear relationship between the applied strain and the resulting focal length (Fig. 6c, Table S6). Quad-axis actuation achieved a total tuning range of 5.62 mm (Δ*f* ∙ *f*_0_^−1^ = 11.8%; slope = 29.7), significantly exceeding the 1.77 mm range achieved under uniaxial loading. This effective tunability, superior to conventional elastomeric lenses such as PDMS^25,26^, arises from the high refractive index of IR-FCh-60 (*n* ≥ 2.139; Supplementary Fig. S7f), which exceeds that of typical DL materials (*n* ≤ 1.52)^24,37^; gray region, Supplementary Fig. S7g. To further contextualize the device performance, a comparative table (Supplementary Table S8) has been included, listing key metrics (aperture, tuning range, PV value, spectral range, and actuation mechanism) of our IR-DL alongside representative liquid lenses and polymer-based DLs. This comparison demonstrates that our IR-DL is the only deformable lens currently capable of operating across an ultra-broadband IR spectral range, with focal length adjustment remaining effective across NIR, MWIR, and LWIR bands (Fig. 6d).

Imaging experiments further evaluated the potential of IR-DL for IR adaptive optics (Supplementary Fig. S7h). For a plano-convex lens, at zero strain, the lens produced high-resolution IR images (Fig. 6e). Under tensile strain, the focal plane shifted backward, resulting in a minified, blurred image with an expanded field of view; compressive strain, conversely, yielded a magnified image with a constricted field of view (Supplementary Movie S6). The image quality was fully restored upon force release, confirming reversible wavefront modulation. In a plano-concave setup, multi-object imaging at varying distances demonstrated active focal tuning and depth-of-field modulation (Fig. 6f, Supplementary Movie S7). In the relaxed state, the distant object “NBU” was sharply imaged. Upon stretching, focus shifted to the nearer object, “duck”. Releasing strain restored focus to the original target, illustrating real-time focal switching and depth selectivity. These results highlight the capability of IR-DLs to dynamically adjust focal length, field of view, and depth of field through mechanical strain. The reversible and controllable optical tuning offered by these chalcogenide-glass-based lenses presents significant potential for adaptive IR imaging systems, including tunable short- and long-range applications.

## Discussion

In this work, we present S_60_Se_40_ (IR-FCh-60), an IR-transparent and ultra-flexible chalcogenide glass that overcomes the classic trade-off between IR transparency and mechanical compliance. This material simultaneously achieves an ultralow Young’s modulus (*E* ≈ 0.0037 GPa), exceptional tensile strain (~650%), efficient strain recovery (~80%), and room-temperature self-healing capability, while maintaining broadband IR transmission up to 21 μm. The ultralow phonon energy afforded by its heavy-atom components (S and Se) suppresses multi-phonon absorption, extending IR cutoff, while covalent bonding determines the short-wave cutoff -collectively yielding a broad atmospheric transmission window (0.62–21 μm). A physically cross-linked DN structure confers polymer-like mechanics. This structure comprises covalent [Se, S]_n_ chains interwoven with dynamic S/Se rings, enabling an ultralow modulus and large deformations through reversible chain straightening/slippage and bond exchange. In situ Raman spectra confirm the reversible elongation of Se–Se bonds during deformation, underpinning its exceptional elasticity behavior. Autonomous self-healing is mediated by high chain-ring mobility and dynamic bond exchange (S–S, Se–Se, Se–S). We demonstrate the material’s applicability through scalable fabrication of IR-DLs, highlighting their potential for adaptive optics, bioimaging, and metrology. This study establishes a new class of IR materials combining glass-like transparency and polymer-like flexibility, with broad implications for next-generation adaptive IR technologies.

## Materials and methods

### Synthesis of polymer-like IR-FCh glasses

Chalcogenide glasses with compositions S_*x*_Se_100-*x*_ (*x* = 0, 30, 45, and 60, molar ratios) were synthesized via vacuum melt-quenching. Remarkably, glasses with high sulfur content (*x* = 30, 45, and 60) exhibited polymer-like ultra-flexibility and a broad transmission window, leading to their designation as IR ultra-flexible chalcogenide (IR-FCh) glasses specifically IR-FCh-30, IR-FCh-45, and IR-FCh-60. The synthesis procedure was followed: High-purity sulfur (99.999%, Aladdin) and selenium (99.999%, Aladdin) were weighed (40 g total) according to stoichiometric ratios, loaded into a quartz tube, and flame-sealed under vacuum (≈10^−3^ Pa). The sealed quartz tube was heated in a rocking furnace to 673 K at 0.8 K min^−1^ and held for 48 h for homogenization. The melt was water-quenched to obtain a cylindrical glass ingot, which was then sectioned into 2 mm-thick wafers and polished to optical quality.

### Characterizations

The structural analyses of glasses were investigated by Micro-Raman spectra on a Raman spectrometer (Renishaw in Via, London, UK) using a 785 nm laser source for excitation. Transmission spectra of the samples were obtained using a Perkin-Elmer-Lambda 950 UV/VIS/NIR spectrophotometer (250–2500 nm range) and a Nicolet 380 Fourier-transform IR spectrometer (2.5–25 μm range). Refractive dispersion curves of the glasses were measured by using a J.A. Woollam IR-VASE spectroscopic ellipsometry with a measurement precision of ±0.0005.

Room-temperature compression, three-point bending, tensile, and tensile relaxation tests were conducted on a horizontally placed mechanical testing machine ZW990LA (Dongguan Zhiqu Precision Instrument Co., Ltd). For compression, ϕ6 × 10 mm cylindrical samples were used; the loading speed was set at 0.1 mm min^−1^. For the three-point bending, the specimen size was 20 × 3 × 1.5 mm (length × width × thickness), and the span was set to 10 mm; the loading speed was set at 0.1 mm min^−1^. For tension testing and tensile relaxation testing, bar-shaped samples (52 × 15 × 1.1 mm) were further machined into bone-shaped specimens with spans of 30 × 5 × 1.1 mm; the loading speeds were set at 5 mm min^−1^ and 1 mm min^−1^, respectively. DMA was conducted on a DMA850 instrument in tension mode with a temperature ramp rate of 4 °C min^−1^ at 6.2 Hz. All mechanical and optical tests were conducted under ambient conditions at ambient laboratory atmosphere without the use of an inert environment.

### Simulation details

Atomic models of pure Se, IR-FCh-30, and IR-FCh-60 glasses were constructed in Materials Studio 2023, annealed, and subjected to tensile deformation using Large-scale Atomic/Molecular Massively Parallel Simulator software (LAMMPS 2023)^47,48^. Structural characterization, including measurements of bond lengths, bond angles, and the temporal evolution of molecular chains and ring structures, was conducted using the OVITO software package^48^. The pure Se model contained 21600 atoms in a cubic cell of 89.13 × 89.13 × 89.13 Å; IR-FCh-30 and IR-FCh-60 models each contained 21688 atoms in cells of 87.28 × 87.28 × 87.28 Å and 83.92 × 83.92 × 83.92 Å, respectively. The samples were first melted and maintained at 670 K for 0.5 ns, a time span that is sufficiently long to equilibrate the liquids. Subsequently, the melts were cooled down to 300 K for 0.5 ns to obtain disordered amorphous glasses^40^. All the simulations were carried out in the isothermal-isobaric (NPT) ensemble under zero pressure and Universal Force Field^47^. Finally, the prepared glass samples were subjected to uniaxial tensile deformation along the *x*-axis at a constant rate of 8 × 10^−8^ nm ps^−1^ (equivalent 5 mm min^−1^), with calculations ceased upon reaching a strain of 500%. The relationship between bond lengths, bond angles, and tensile strain was statistically analyzed using Python to call the relevant modules in OVITO. All simulations were carried out using LAMMPS with a time step of 0.1 fs.

### Design and fabrication of the IR-DL

The IR-DL (Supplementary Fig. S7a) was fabricated from IR-FCh-60 material (in this work: S_60_Se_40_) with an initial diameter (*D*) and radius of curvature (*R*_*0*_). Eight metal anchors were embedded in the IR-DL. The metal anchors also define the clear aperture (*d*_0_), which is the distance between the two vertices of the opaque anchors at zero strain. The focal length of IR-DL may be varied by application of strain. When a force, *F*_f_, is applied radially to the metal anchors, these move outward while the counterforce (*F*_e_) is provided by the Young’s modulus (*E* = 3.77 MPa). While straining the IR-DL in the equatorial plane (Poisson’s ratio *v* = 0.38 > 0), the radius of curvature increases and so does the focal length of the lens. The strain, *ϵ*, which uses the initial clear aperture *d*_0_ of the lens as a reference, is expressed as^25^

1 $$\epsilon =\Delta d/{d}_{0}$$

where Δ*d* is the diameter increment due to the movement of the metal anchors.

The plano-convex and plano-concave IR-DLs (*d*_0_ = 31 mm, *D* = 45 mm) were fabricated by the melt pouring molding method (Supplementary Fig. S7b). Initially, eight metal T-shaped anchors (thickness = 2 mm; height = 4 mm; width = 10 mm) were aligned symmetrically in four diametrical pairs in the designated slots of the predesigned graphite mold, 5 mm from the edge. Prefabricated IR-FCh-60 glass was melted at 300 °C for 30 min under N₂ atmosphere, poured into the mold, and quenched. The resulting lens was optically polished and integrated with an eight-actuator drive system to form the final electrically tunable IR-DL. Initial properties of lenses are summarized in Supplementary Table S5.

### Optical metrology of the IR-DL

Transmitted wavefronts were measured using a self-built optical bench equipped with a wavefront sensor (WFS), as depicted in Fig. 6a. The IR-DL’s surface profiles were characterized with a Mach-Zehnder interferometer (HMB 150MWIR) with a 3.39 µm laser source. Surface wavefront aberrations were fitted using the first 36 Zernike polynomials. Measurements used an 8 mm aperture, 30 Hz acquisition, 3 frame averaging, and 30 ms exposure. All tests were performed at room temperature.

## Supplementary information

Supplementary Information

Supplementary Movie S1

Supplementary Movie S2

Supplementary Movie S3

Supplementary Movie S4

Supplementary Movie S5

Supplementary Movie S6

Supplementary Movie S7

## Data Availability

All data are available in the main text or the Supplementary Materials. Correspondence and requests for materials should be addressed to the corresponding authors.
