# Supplementary material for: An infrared-transparent flexible glass for adaptive optics
Source: Light Sci Appl. 2026 Jul 30;15:334. doi: 10.1038/s41377-026-02409-z (PMC13424142; doi:10.1038/s41377-026-02409-z)
Supplement: Supplementary file 1 — Supplementary Information [file 41377_2026_2409_MOESM1_ESM.docx]

Supplementary Information for

**An Infrared-Transparent Flexible Glass for Adaptive Optics**

Saihui Li^1,2,3^, Linling Tan^1,2,3^*, Jianqiang Ma^4^, Shiliang Kang^1,2,3^, Chengwei Gao^1,2,3^, Shixun Dai^1,2,3^, Changgui Lin^1,2,3^*

*^1^Laboratory of Infrared Materials and Devices, The Research Institute of Advanced Technologies, Ningbo University, Ningbo 315211, China*

*^2^Key Laboratory of Photoelectric Detection Materials and Devices of Zhejiang Province, Ningbo 315211, China*

*^3^Engineering Research Center for Advanced Infrared Photoelectric Materials and Devices of Zhejiang Province, Ningbo 315211, China*

*^4^College of Mechanical Engineering and Mechanics, Ningbo University, Ningbo 315211, China*

**Corresponding Emails (telephone number):** [*tanlinling@nbu.edu.cn*](mailto:tanlinling@nbu.edu.cn) (18813759342)*;* [*linchanggui@nbu.edu.cn*](mailto:linchanggui@nbu.edu.cn) (1888106417)

**This PDF file includes:**

Figures S1 to S7

Tables S1 to S8

Movies S1 to S7

Legends for Movies S1 to S7

**Figures**


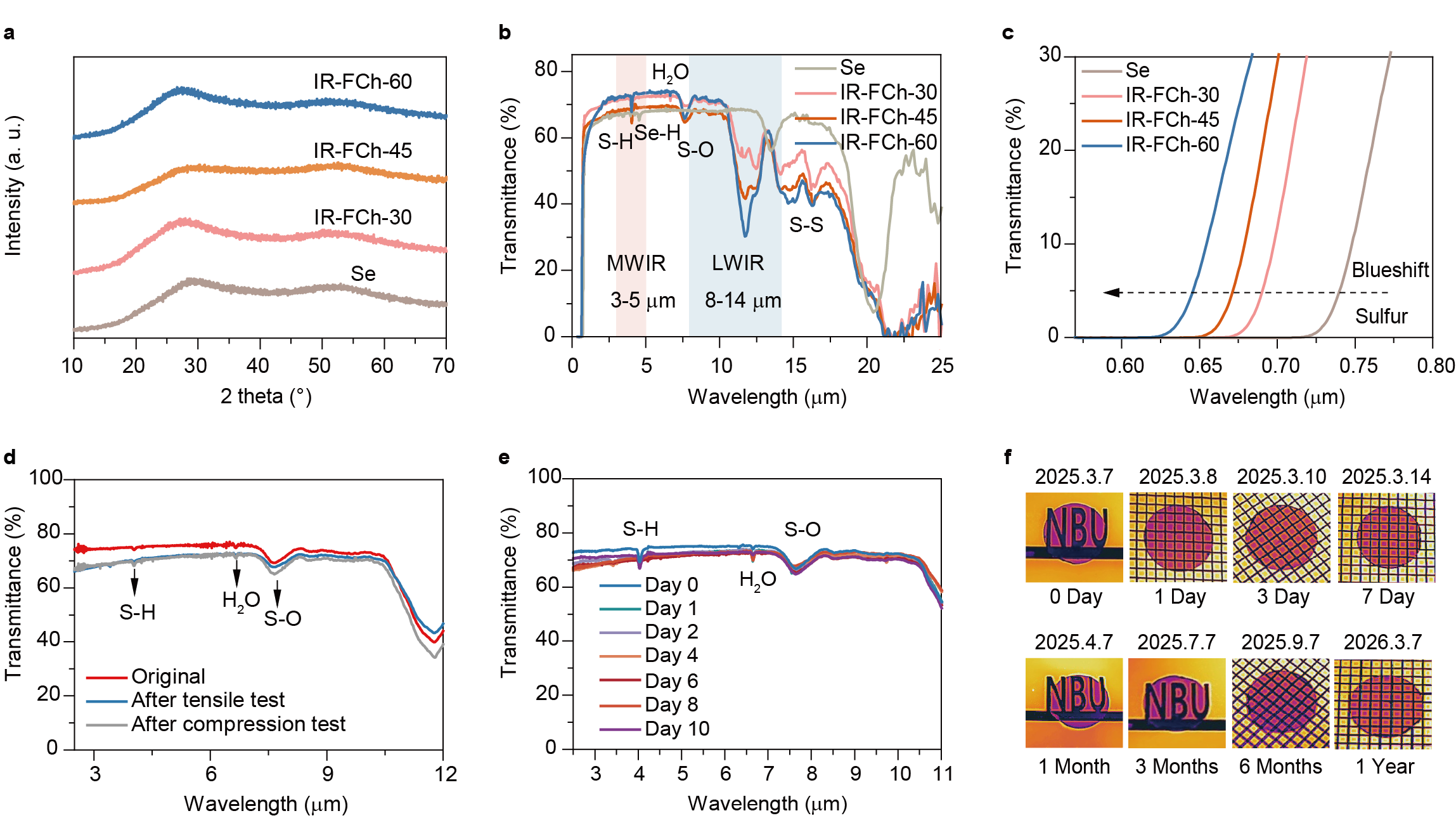


**Fig. S1** **Ultrabroad IR transmission of IR-FCh glasses.** (a) XRD patterns, (b)Transmission spectra and (c) short-wavelength cut-off characteristics of IR-FCh and pure Se glasses. Theses IR-FCh glasses and pure Se glass are all amorphous. The ultralong IR cut-off edges (~21 µm for IR-FCh; >25 µm for Se) originate from multi-phonon absorption processes dominated by Se-Se bond vibrations. Introducing sulfur blue-shifts the short-wavelength cut-off edge from 0.72 (Se) to 0.62 µm (S_60_Se_40_). Characteristic impurity absorptions are assigned to: S-H (4.0 µm), Se-H (4.6 µm), H_2_O (6.6 µm), S-O (7.7 µm), and S-S stretching in chains and rings (11.8, 14.1 and 16.4 µm). Those impurity-related absorptions can be further reduced through purification by a combined strategy of chemical reaction and distillation. All compositions exhibit high transmittance across critical atmospheric windows (8-14 µm, 3-5 µm, and 1-3 µm), with sulfur enrichment improving performance in the 1-15 µm spectral range. Impurity effects are attributable to raw materials and atmospheric exposure and may be attenuated through advanced glass purification. (d) Transmittance spectra of IR-FCh-60 glass before and after mechanical testing. (e) Transmittance spectra of IR-FCh-60 glass after environmental exposure for 10 days. No significant increase in the S-O peak intensity is observed, suggesting that no oxidation occurred during mechanical testing and environmental exposure. (f) The LWIR images (8-14 μm) of IR-FCh-60 at different environmental exposure times. The sample retains excellent IR transparency even after one year of storage in air. These results demonstrate that the IR-FCh-60 material possesses reasonable oxidation resistance.


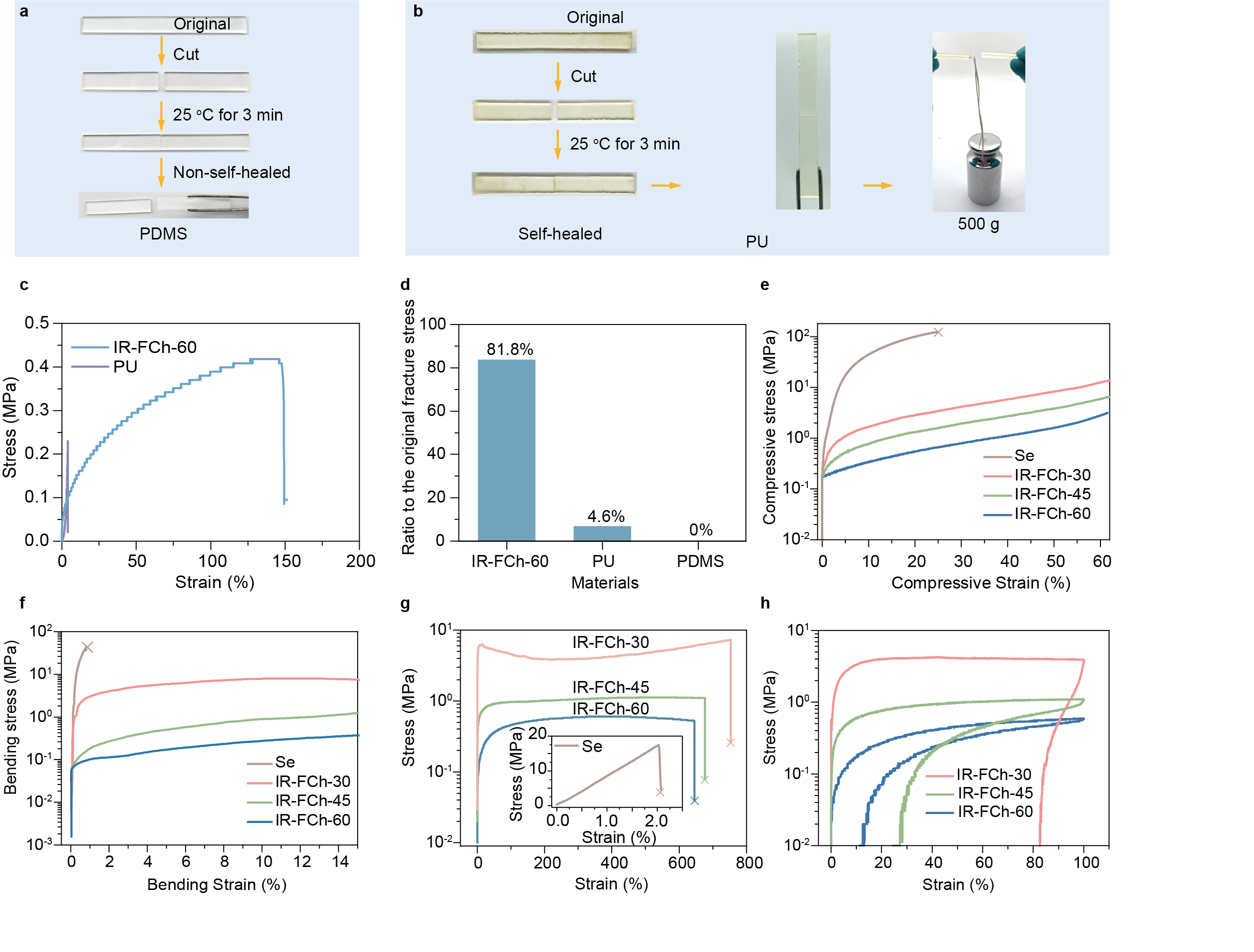


**Fig. S2 Room-temperature self-healing for IR-FCh-60, PDMS, and PU, and extreme deformability of IR-FCh glasses.** Self-healing behavior of PDMS (a) and PU (b) at room temperature. Bar-shaped samples (40 mm × 5 mm × 1.5 mm, length × width × thickness) were sectioned into two parts and subsequently brought into contact for 3 minutes. The IR-FCh-60 (shown in Fig. 2c IV, Supplementary Movie S5) exhibited remarkable self-healing capability, readily supporting a 500 g weight after rejoining. In contrast, self-healed PU could only sustain its own weight, and PDMS exhibited virtually no self-healing ability under identical conditions (Supplementary Movie S5). (c) Tensile stress-strain curves of IR-FCh-60 and PU after healed 3min under room temperature. (d) The ratio of the fracture stress of IR-FCh-60, PU and PDMS after healed 3min under room temperature. (e-g) Stress-strain curves of IR-FCh and pure Se glasses for compression (e), bending (f) and tension (g) tests. All strain-stress curves exhibit a short elastic deformation stage and then turn to a long plastic stage for IR-FCh glasses, whereas pure Se glass shows limited ductility. In compression test (e), IR-FCh glasses withstand over 60% strain without fracture, while pure Se glass bursts instantly at 28.3% strain. In the three-point bending test (f), the IR-FCh glasses endure bending strains exceeding 15% without cracking, whereas the pure Se glass fractures at a minor strain (1.2%). Note: IR-FCh samples did not reach the fracture strain, and the samples did slip and squeeze with the fixture. Under tension test (g), IR-FCh glasses achieve strains of ~650-700%, far exceeding the ~2% strain of pure Se glass. (h) Tensile relaxation tests of IR-FCh glasses at 100% strain. Sulfur enrichment reduces modulus while enhancing shape recovery, positioning IR-FCh-60 as the optimal composition for polymer-like mechanical performance.


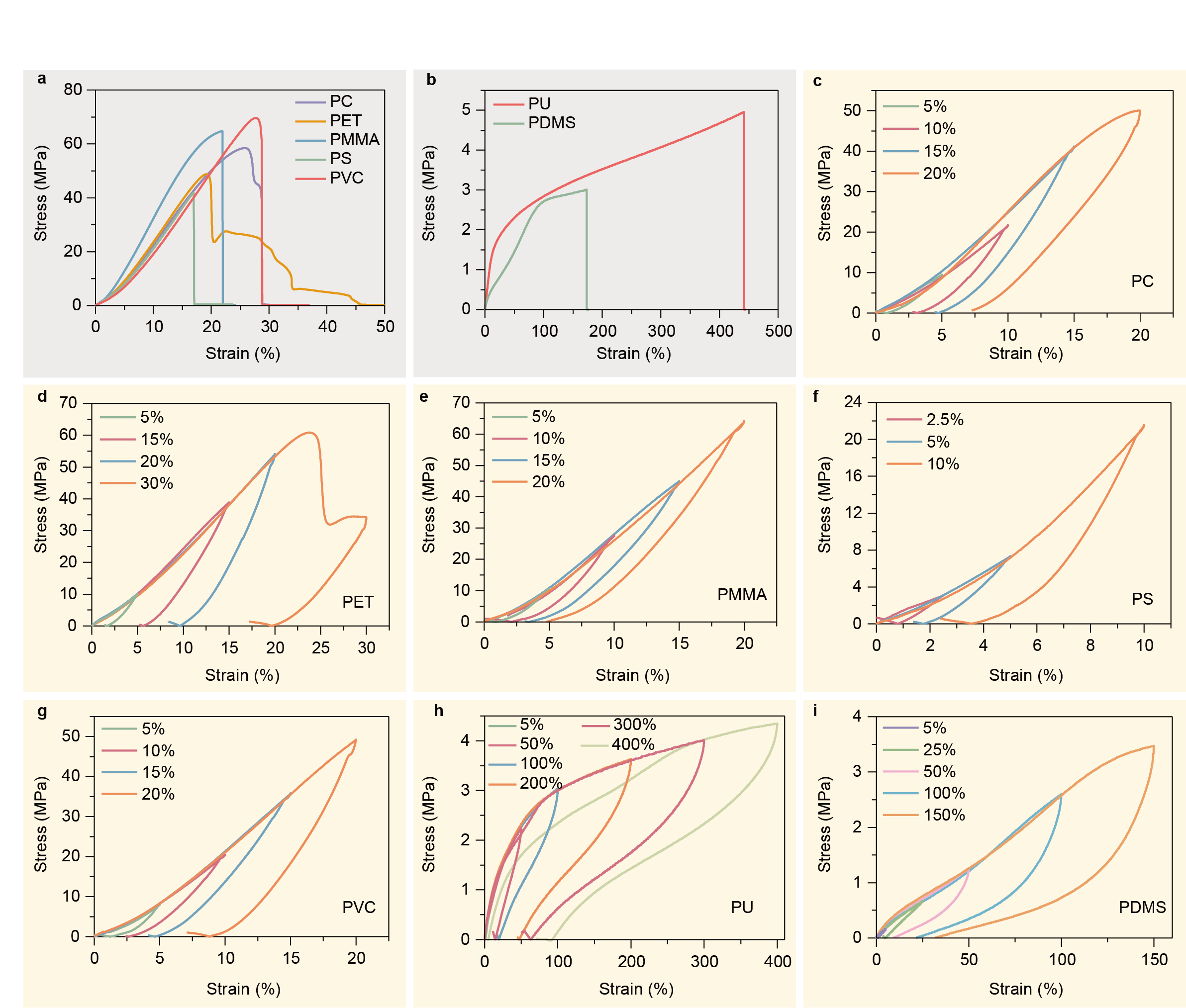


**Fig. S3 Stress-strain curves of organic materials.** (a, b) Stress-strain curves of the conventional organic materials under tension at room temperature, including rigid materials (PC, PET, PMMA, PS and PVC) and flexible materials (PDMS and PU). The low-modulus PU and PDMS exhibited high flexibility, with maximum tensile strains of approximately 180% and 440%, respectively. In contrast, high-modulus polymers (e.g., PC, PET, PMMA, PS, and PVC) showed rigid mechanical behavior, achieving only limited tensile strains (≤ 45%) even under high applied force. (c-i) Tensile stress relaxation tests for PC, PET, PMMA, PS, PVC, PU and PDMS at various strains. High-modulus organic materials (PC, PET, PS, and PVC) exhibited significant plastic deformation with recovery ratios below 77% before fracture (at tensile strain < 50%), while PMMA displayed weaker plasticity with a recovery ratio near 80%. The ultralow-modulus elastomers PDMS and PU demonstrated pronounced recovery ability (recovery ratios ~80%) across wide tensile strain ranges (PDMS: 5-150%; PU: 5-400%).


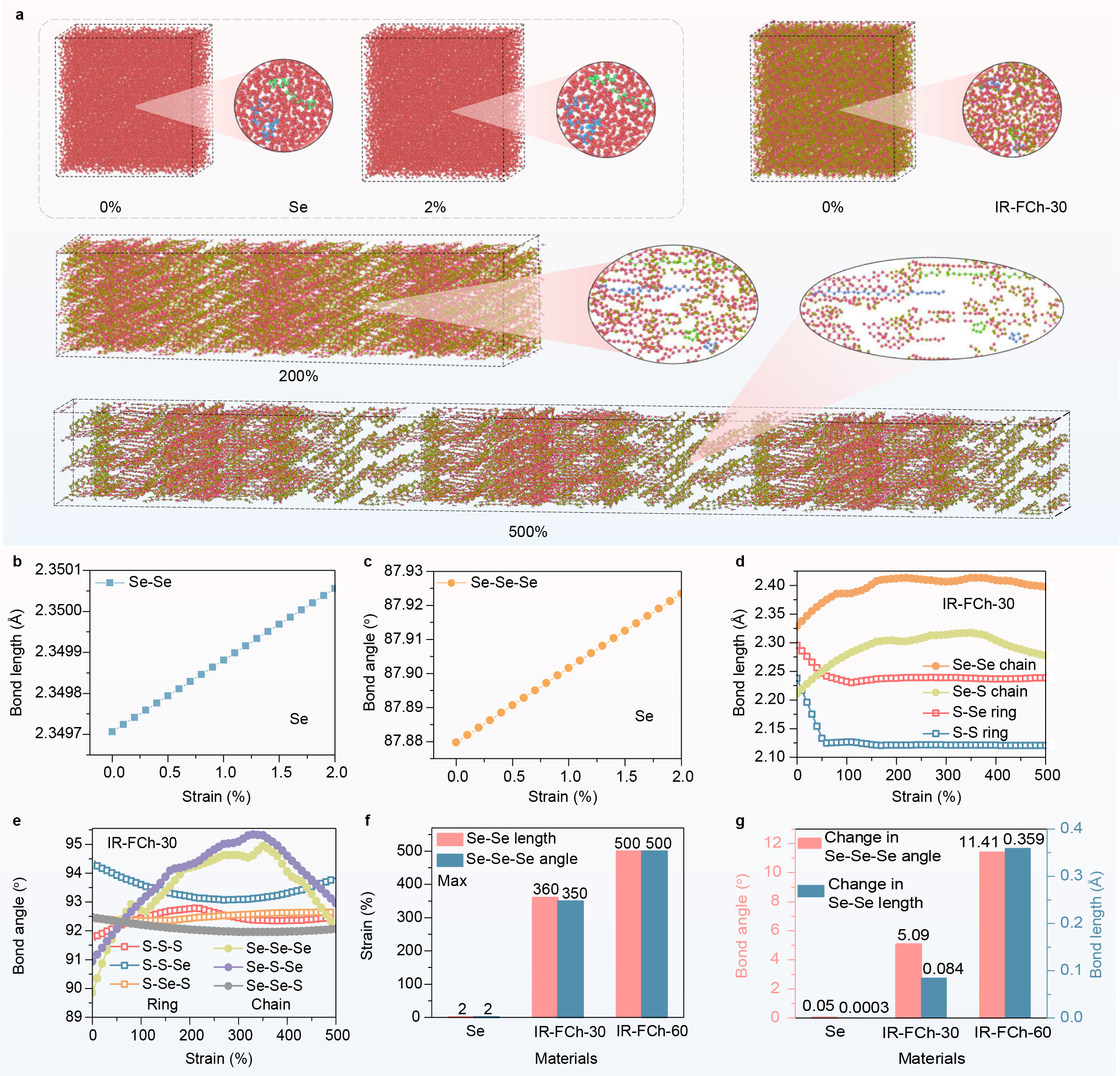


**Fig. S4** **Origin of the polymer-like ultrahigh flexibility.** (a) Atomic snapshots and cross-sections of pure Se (0%, 2% strain) and IR-FCh-30 (0%, 200%, 500% strain) under tension deformation. Chain (blue) and ring (green) motifs are highlighted to illustrate structural evolution. Evolution of bond length (b, d) and bond angles (c, e) of chain and ring structural units as a function of tensile strain. In pure Se glass, both Se-Se bond lengths and Se-Se-Se bond angles in pure Se glassy chain-like structures increase monotonically with increasing tensile strain. In contrast, IR-FCh-30 exhibits an initial increase followed by a decrease in Se-Se/Se-S bond lengths and Se-Se-Se/Se-S-Se bond angles. Maximum bond parameter values occur at 2% strain for pure Se glass, and at 350% and 500% strain for IR-FCh-30 and IR-FCh-60 glasses, respectively. The tensile strains corresponding to the peak/maximum values of the Se-Se bond lengths and Se-Se-Se bond angles in the long-chain structures of pure Se, IR-FCh-30 and IR-FCh-60 glass (f), as well as the maximum changes in these bond lengths and bond angles (g). The maximum variations in Se-Se-Se bond angles expand from 0.05 ° to 11.41 °, while Se-Se bond length changes rise from 0.018 to 0.359 Å with increasing the content of sulfur. Sulfur enrichment amplifies bond distortion capacity, enabling extreme deformability through reversible covalent bond elongation.


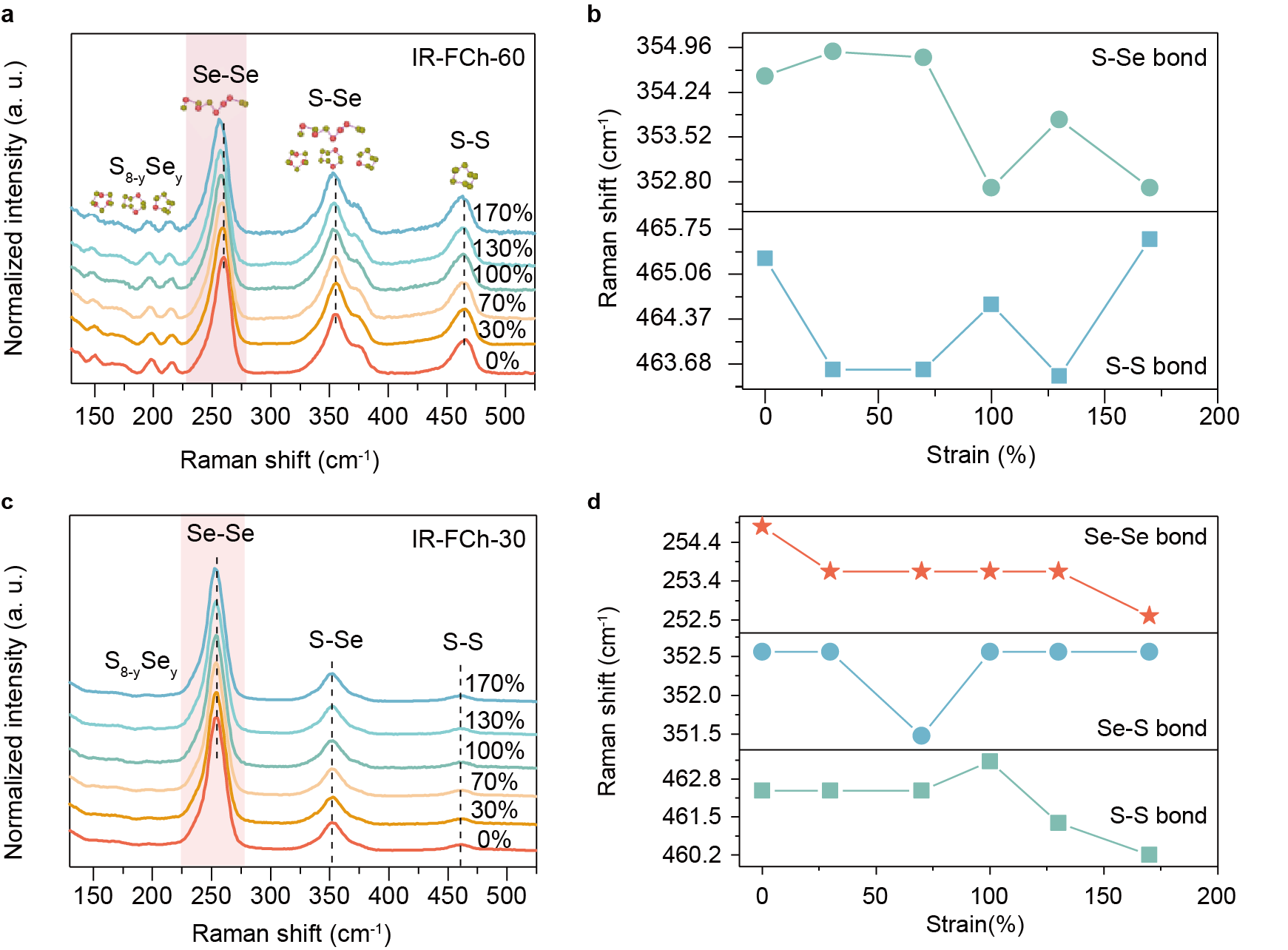


**Fig. S5.** **Strain-dependent bond evolution via in situ Raman spectroscopy.** (a, c) Raman spectra of IR-FCh-60 (a) and IR-FCh-30 (c) under tensile strain. A robust band around 260 cm^-1^ corresponds to the symmetric stretching mode of the Se-Se bond within [Se]_n_ chains. The band near 465 cm^-^¹ is assigned to symmetric S-S stretching in S_8_ rings, while features at ~355 and ~379 cm^-^¹ are ascribed to S-Se vibrations in both chains and rings. Furthermore, the spectrum of IR-FCh-60 exhibits bands between 160-210 cm^-1^, with peaks at 202, 175, and 163 cm^-1^ aligning well with bending modes of S_7_Se_1_, S_6_Se_2_, and S_5_Se_3_ rings, respectively. Notably, for IR-FCh-30, the shoulder peak at ~379 cm⁻¹ and the bands in the 160 to 190 cm^-1^ are nearly undetectable, due to the low sulfur content. (b, d) Strain-dependent frequency shifts of S-Se and S-S bonds in IR-FCh-60 (b) and Se-Se, S-Se and S-S bonds in IR-FCh-30 (d). Stepwise tensile loading induces a pronounced redshift of the Se-Se stretching band in the long-chains (4 cm^-^¹ for IR-FCh-60; 2.5 cm^-^¹ for IR-FCh-30). Uniaxial strain elongates the bond lengths and widens the bond angles, increasing interatomic distances and reducing force constants, thereby lowering the vibrational frequencies. In contrast, Raman bands arising solely from S-S bonds within eight-membered rings exhibit irregular and markedly smaller shifts; this reflects the fact that the eight-membered rings undergo only minor deformation under tension. These results are corroborated by molecular-dynamics simulations, which indicate that deformation under tension occurs primarily in the chains rather than the rings. Rectangular strip samples (length × width = 10 mm × 5 mm, thickness ≈ 10 µm) were used.


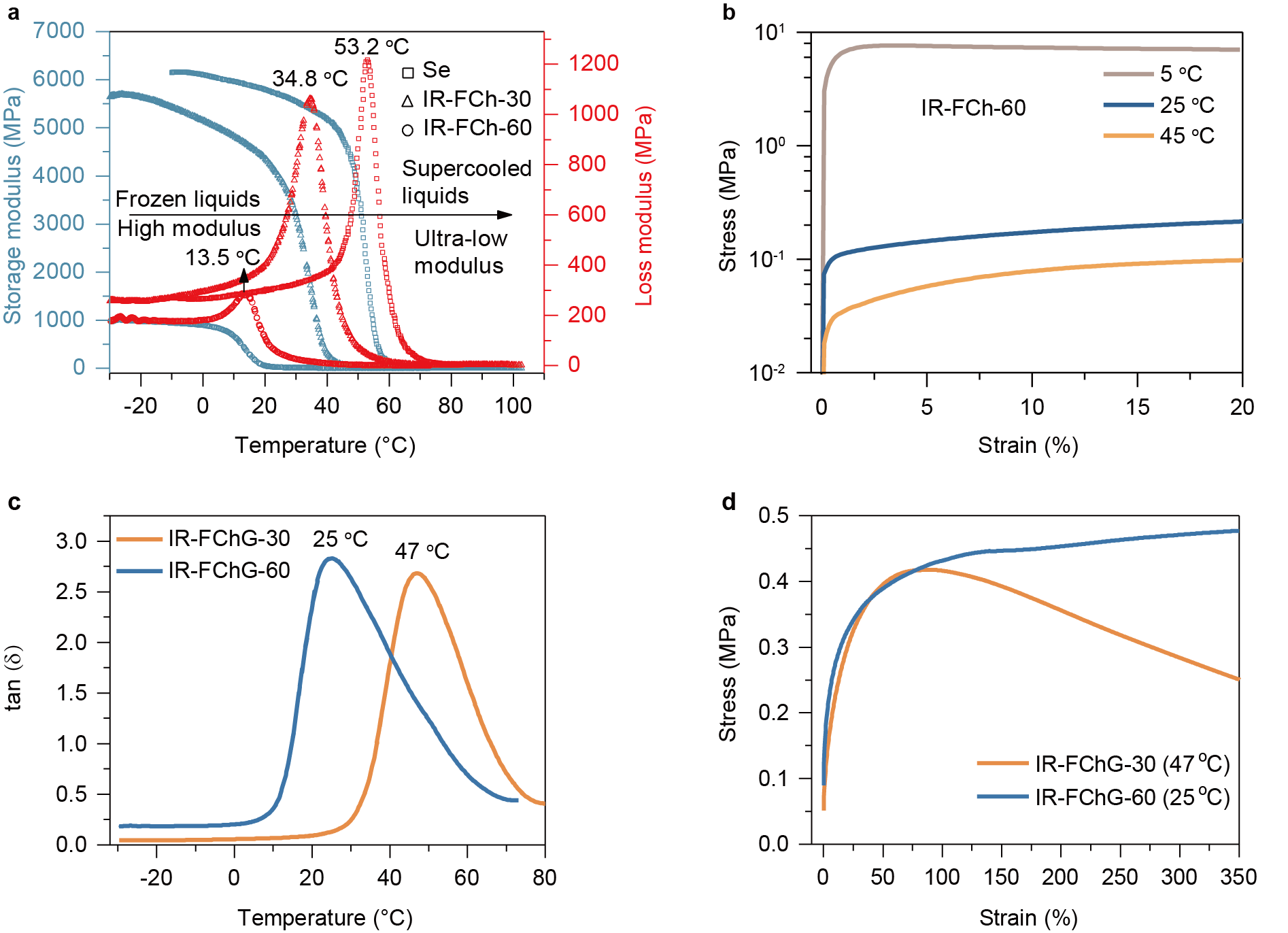


**Fig. S6 Origin of the polymer-like ultralow modulus at room temperature.** (a) Dynamic mechanical analysis of pure Se glass, IR-FCh-30 and IR-FCh-60 glasses, showing storage modulus and loss modulus as functions of temperature. As the temperature rises, the storage modulus begin to decrease and the loss modulus (measuring the energy dissipation) of IR-FCh-30 and IR-FCh-60 start to rise and peak at 34.8 °C and 13.5  °C, respectively, indicating that the glass transition processes (*T*_g, IR-FCh-30_=34.8 °C and *T*_g,_ _IR-FCh-60_ = 13.5  °C, signified by the peak of the loss modulus) occur nearly or below room temperature. Their modulus all dropped sharply in the glass transition region, especially IR-FCh-60, which exhibited an ultra-low modulus (*E* _IR-FCh-60_ = 16 MPa) at room temperature. In contrast, pure Se glass demonstrates a high storage modulus of 5676 MPa at 25 °C with a loss modulus peak at 53.2 °C, indicating a glass transition event occurs at this elevated temperature (significantly exceeding room temperature). ‘Supercooled liquid’ and ‘frozen liquid’ refer to the states of a glass-forming liquid when it is below and above the *T*_g_, respectively. (b) Stress-strain curves of IR-FCh-60 in the ‘supercooled liquids’ and the rigid ‘frozen liquids’ state, tested at 45 ℃, 25 ℃ and 5 ℃, respectively. (c) Loss tangent δ which is the ratio of storage modulus to loss modulus in DMA tests at various temperatures of IR-FChG-30 and IR-FChG-60 materials. (d) The tensile stress-strain behavior of IR-FCh-30 and IR-FCh-60 under the condition where the temperature was consistently maintained 12 °C above their respective *T*_g_ values and aligned with their respective loss factor (tan δ) peaks.





**Fig. S7** **Fabrication and optical properties of IR-DL.** (a, b) Design (a) and fabrication (b) of IR-DL. (a) Schematic of the lens geometric configuration with eight metallic T-shaped anchors viewed along the optical axis (upper panel), and conceptual design and structural schematic of the electronic driver for deformation control (lower panel). Strain is applied via eight T-shaped anchors. The relaxed-state dimensions (diameter, clear-aperture and thickness) and optical properties (focal length, radius of curvature) of the IR-DL are widely tunable; lens type (concave/convex) can be customsized via mold design, allowing on-demand reconfiguration. (b) Fabricated plano-convex and plano-concave IR-DLs (45 mm diameter, 31 mm clear aperture), demonstrating programmable IR optics. The minimum clear aperture and diameter are limited only by the anchor contact area. (c, d) Surface quality characterization of the as-fabricated plano-convex IR-DL via interferometer. Interferogram (c) and wavefront map (d) of the reflective surface. (e) Wavefront profiles of the plano-convex IR-DL at -3.6%, 0%, 5.3%, 10.7% and 15.1% tensile strain at 850 nm. (f) Refractive index at various wavelengths of IR-FCh-60 glass. The value at 850 nm was derived from focal length measurements of the relaxed IR-DL. (g) Refractive index comparison between IR-FCh-60 and conventional deformable lens materials (liquids and organic elastomers). (h) Schematic of the long-wave IR (LWIR) imaging setup. For the plano-convex lens, a heated “F_F_”-patterned PMMA mask (opaque in LWIR) served as the target (Fig. 6e, Supplementary Movie S6). For the plano-concave lens, a metallic "NBU" sign and a duck toy filled with hot water, separated by 6 cm, were used as the far- and near-distance targets, respectively, for multi-object imaging with the plano-concave lens. A thermal camera (LWIR, 8-14 μm transmission range) was used for imaging, with the IR-DL positioned between the object and the camera.

**Tables**

**Table S1.** Data source for mechanical properties of typical IR optical materials and organic optical materials used in Fig. 2a. IR optical materials include crystals (MgF_2_, ZnSe, Ge, BaF_2_, CaF_2_) and chalcogenide glasses (Ge_28_Sb_12_Se_60_, As_2_Se_3_ and As_2_S_3_). Organic optical materials include polystyrene (PS), polyvinyl chloride (PVC), polyethylene glycol terephthalate (PET), polycarbonate (PC), polymethyl methacrylate (PMMA), polyurethane (PU), DBA, Polytetrafluoroethylene (PTFE) and polydimethylsiloxane (PDMS).

| Materials system | Young’s modulus, *E* (GPa) | Elastic compliance, 1/*E* | IR cutoff edge (µm) | References of Young’s modulus | References of IR cutoff edge |
| --- | --- | --- | --- | --- | --- |
| IR-FCh-60 | 0.0037 | 265.2519 | 21.00 | This work | This work |
| Ge | 102.7000^†^ | 0.0097 | >25^†^ | Crystran,  https://www.crystran.com/optical-materials/germanium-ge | Crystran,  https://www.crystran.com/optical-materials/germanium-ge |
| BaF_2_ | 53.0700^†^ | 0.0188 | 11.85^†^ | Crystran,  https://www.crystran.com/optical-materials/barium-fluoride-baf2 | Crystran,  https://www.crystran.com/optical-materials/barium-fluoride-baf2 |
| CaF_2_ | 146.0000^†^ | 0.0068 | 12.07^†^ | Cornin, https://www.corning.com/media/worldwide/csm/documents/Corning_AdvancedOptics_OpticalGradeCaF2_DataSheet.pdf | Thorlabs, https://www.thorlabs.com/newgrouppage9.cfm?objectgroup_id=3978 |
| MgF_2_ | 138.0000^†^ | 0.0072 | 10.01^†^ | Corning,  https://www.corning.com/media/worldwide/csm/documents/MgF2%20PI%20sheet.pdf | Thorlabs, https://www.thorlabs.com/NewGroupPage9.cfm?ObjectGroup_ID=5582 |
| ZnSe | 67.2000^†^ | 0.0148 | 21.85^†^ | Crystran,  https://www.crystran.com/optical-materials/zinc-selenide-znse | Crystran,  https://www.crystran.com/optical-materials/zinc-selenide-znse |
| As_2_S_3_ | 15.9000^†^ | 0.0628 | 12.61^†^ | *Sens. Actuators, B* **175**, 142-148, (2012). | *Sens. Actuators, B* **175**, 142-148, (2012). |
| As_2_Se_3_ | 18.5000^†^ | 0.0540 | 19.85^†^ | *Optik* **245**, 167693, (2021). | *Opt. Express* **23**, 23472-23483, (2015). |
| Ge_28_Sb_12_  Se_60_ | 22.1000^†^ | 0.0452 | 20.50^*^ | Schott, www.schott.com/advanced_optics | / |
| DBA | 5.4000 | 0.1851 | 2.18^†^ | *Angew. Chem. Int. Ed.* **62**, e202217329, (2023). | *Opt. Lett.* **47**, 509-512, (2022). |
| PMMA | 3.0000^*^, 3.2000^†^ | 0.3333^*^ | 4.80^*^, 5.21^†^ | Matmake, https://matmake.com/properties/youngs-modulus-of-polymers-and-plastics.html | Femtum, https://femtum.com/resources/laser-processing-of-polymer-in-the-mid-infrared/ |
| PS | 2.3000^*^, 1.9000-2.9000^†^ | 00.4347^*^ | 6.50^*^ | Matmake, https://matmake.com/properties/youngs-modulus-of-polymers-and-plastics.html | / |
| PET | 3.2000^*^, 3.0000-11.000^†^ | 0.3125^*^ | 2.70^*^ | The Engineering Tool Book,  https://www.engineeringtoolbox.com/young-modulus-d_417.html | Matmake; https://femtum.com/laser-processing-of-polymer-in-the-mid-infrared/ |
| PC | 1.8000^*^, 2.3000-2.4000^†^ | 0.5555^*^ | 4.10^*^, 2.30^†^ | Matmake, <https://matmake.com/properties/youngs-modulus-of-polymers-and-plastics.html> | *Lasers Manuf. Mater. Process* **6**, 113-125, (2019). |
| PVC | 2.2000^*^, 2.1000-2.7000^†^ | 0.4545^*^ | 5.70^*^ | Matmake, https://matmake.com/properties/youngs-modulus-of-polymers-and-plastics.html | / |
| PDMS | 0.0016^*^, 0.0015^†^ | 625.00^*^ | 5.60^*^, 5.96^†^ | *Procedia Struct. Integrity* **37**, 383-388, (2022). | *Sci. Rep.* **8**, 9132, (2018). |
| PU | 0.0037^*^, 0.0025^†^ | 270.2700^*^ | 2.20^*^ | *Modern Approaches on Material Science* **2**, 251-255, (2020). | / |
| PTFE | 0.4000 | 2.500 | / | The Engineering Tool Book,  https://www.engineeringtoolbox.com/young-modulus-d_417.html | / |

* Measured in this work; † from the references.

**Table S2.** Data source for fatigue property of organic optical materials and IR-FCh-60 used in Fig. 3a. Organic optical materials include PS, PMMA, PC, PVC, PET, PDMS and PU.

| Materials | IR-FCh-60 | PS | PMMA | PC | PVC | PET | PDMS | PU |
| --- | --- | --- | --- | --- | --- | --- | --- | --- |
| Maximum tensile strain (%) | 647 | 17 | 22 | 29 | 29 | 45 | 173 | 441 |

**Table S3.** Data source for fatigue property of organic optical materials and IR-FCh-60 glass used in Fig. 3b. Organic optical materials include PS, PMMA, PC, PVC, PET, PDMS and PU.

| Materials | Strain (%) / Recovery rate (%) | | | | | |
| --- | --- | --- | --- | --- | --- | --- |
| IR-FCh-60 | 25/84 | 100/82 | 200/79.2 | 300/78.4 | 400/78.1 | 500/73 |
| PU | 5/84 | 50/82 | 100/80 | 200/76 | 300/78 | 400/77 |
| PDMS | 5/88 | 25/81 | 50/80 | 100/78 | 150/77.8 | / |
| PS | 2.5/68 | 5/64 | 10/65 | / | / | / |
| PC | 5/83 | 10/69 | 15/68 | 20/67 | / | / |
| PET | 5/68 | 15/63 | 20/53 | 30/34 | / | / |
| PMMA | 5/84 | 10/80 | 15/77 | 20/75 | / | / |
| PVC | 5/76 | 10/74 | 15/59 | 20/55 | / | / |

**Table S4.** The ratios of ring to chain in the IR-FCh glasses and their mechanical properties, such as modulus and recovery rate.

| Materials | Ring-to-chain ratio, (%) | Modulus, (GPa) | Recovery rate with elongation reaches 100%, (%) |
| --- | --- | --- | --- |
| IR-FCh-30 | 12.34 | 0.0111 | 16.9 |
| IR-FCh-45 | 23.53 | 0.0053 | 72.5 |
| IR-FCh-60 | 43.13 | 0.0037 | 88.5 |

**Table S5.** Initial properties of fabricated IR-DLs

| IR-DL | Radius of curvature (mm) | Diameter (mm) | Clear aperture (mm) | Thickness (mm) | Focal length at 850 nm (mm) | PV (µm) | RMS (µm) |
| --- | --- | --- | --- | --- | --- | --- | --- |
| Plano-convex lens | 55 | 45 | 31 | 8 | 47.5 | 1.572 | 0.159 |
| Plano-concave lens | 253 | 45 | 31 | 5 | 219.8 | / | / |

**Table S6.** Tuning ranges of focal length for the IR-DL

| Actuation axis | Slope (mm strain^-1^) | Tuning (mm) |
| --- | --- | --- |
| I | 9.0±0.2 | 1.77 |
| I & III | 22.7±0.3 | 4.28 |
| I & II & III & IV | 29.7±0.1 | 5.62 |

**Table S7.** Data source for fatigue property of the materials that have been reported for the fabrication of deformed lens materials (liquids and organic elastomers) and IR-FCh-60 glass used in Fig. S7g.

| Materials system | | Refractive index, *n* | References |
| --- | --- | --- | --- |
| DN chalcogenide glass | IR-FCh-60 | ≥ 2.139 | This work |
| Liquids | Ultrapure water | 1.330 | *Applied Physics Letters* **94**,3268-3274, (2009). |
|  | Silicon oil | 1.600 | *Opt. Express* **14**, 8031-8036, (2006). |
|  | NOA81 | 1.520 | *Opt. Express* **15**, 11328-11335, (2007). |
| Organic elastomers | PDMS | 1.410 | *Appl. Opt.* **50**, 3268-3274, (2011). |
|  | DBA | 1.434 | *Opt. Lett.* **47**, 447182 (2022). |
|  | SPNIP Gel | 1.455 | *ACS Appl. Mater. Interfaces* **13**, 10397-10408, (2021). |
|  | Acrylic | 1.476 | *Opt. Express* **21**, 8669, (2013). |

**Table S8.** The comparation of the IR-DL with representative liquid and polymer-based DLs in terms of aperture, tuning range, PV value, material transmission range/lens’s operating wavelength, and actuation mechanism.

|  | Materials | Aperture (mm) | Tuning range (mm) | PV (μm) | Spectral range/ operating wavelength (μm) | Actuation mechanism | References |
| --- | --- | --- | --- | --- | --- | --- | --- |
| Solid lens | IR-FCh-60 | 31 | 5.62 | 1.572 | 0.62-21/0.8-14 | Servo-driven | This work |
|  | SPNIPGel | 0.75 | Infinity | -/ | 0.3-0.8/0.55 | Piezoelectric drive | *ACS Appl. Mater. Interfaces* **13**, 10397-10408 (2021). |
|  | PDMS | 25.84/25.54 | ~4 | -/ | /0.54 | Servo-driven | *Appl. Opt.* **50**, 3268-3274 (2011). |
|  | PDMS | 16 | 2.4 | 1.76 | /0.633 | Servo-driven | *Light Sci. Appl.* **2**, e98-e98 (2013). |
|  | Acrylic elastomer | / | ~9.6-37 | -/ | /0.55 | Piezoelectric drive | *Opt. Express* **21**, 8669 (2013). |
| Liquid lens | DBA | 1 | 5.6 | -/ | 0.2-2.3/0.63 | Piezoelectric drive | *Opt. Lett.* **47**, 509-512 (2022). |
|  | Glycerol | 5 | Infinity | -/ | 0.4-1.4/0.5, 1.31 | Mechanical drive | *Opt. Express* **18**, 12430-12435 (2010). |
|  | Water | 3 | Infinity | 0.6 | /0.546 | Piezoelectric drive | *Appl. Phys. Lett* **94**, 221108 (2009). |
|  | Silicon oil | 15 | 5.8 | -/ | /0.633 | Servo-driven | *Opt. Express* **14**, 8031-8036 (2006). |
|  | NOA 81 | 2.75 | ~35 | -/ | /0.55 | Driven by conductive polymers | *Opt. Express* **15**, 11328-11335 (2007) |

**Legends for Movies S1 to S7**

**Movies S1-S3. Polymer-like flexibility with IR transparency of IR-FCh-60.** Visible and IR Movies of bending (Movie S1), compression (Movie S2), and tensile (Movie S3) tests. Custom sample geometries were used for clarity: a cylinder (Φ9 × 15 mm; Movie S1), a rectangular cuboid (15 × 5 × 25 mm; Movie S2), and a flat strip (15 × 5 × 2 mm; Movie S3).

**Movie S4. Room-temperature shape memory of IR-FCh-60.** A strip of IR-FCh-60, pre-programmed into a helical shape, autonomously returns to its original configuration upon release.

**Movie S5. Comparative self-healing capability of IR-FCh-60, PDMS, and PU at room temperature.** After cutting and three minutes of contact, the rejoined IR-FCh-60 strip supports a 500 g weight, outperforming PU (self-weight bearing) and PDMS (no healing).

**Movie S6. Dynamically programmable IR imaging with a plano-convex IR-DL.** The focal length is tuned in real time by actuating the lens curvature. Stretching induces minification with an expanded field of view, while compression generates magnification with a constricted field.

**Movie S7.** **Dynamically programmable IR imaging with a plano-concave IR-DL.** The Movie demonstrates real-time focal-length tuning for selective target focusing among objects at different distances. Under tensile strain, the lens shifts focus from the distant “NBU” object to the nearby “Duck,” which becomes sharply resolved. Releasing the strain restores focus to the distant target, illustrating reversible and controllable depth-selective imaging through strain-mediated focal adjustment.
